# Astrocytes in the adult dentate gyrus—balance between adult and developmental tasks

**DOI:** 10.1038/s41380-023-02386-4

**Published:** 2024-01-04

**Authors:** Nicholas Chalmers, Evangelia Masouti, Ruth Beckervordersandforth

**Affiliations:** Institute of Biochemistry, Friedrich-Alexander-Universität Erlangen-Nürnberg, Erlangen, Germany

**Keywords:** Neuroscience, Cell biology

## Abstract

Astrocytes, a major glial cell type in the brain, are indispensable for the integration, maintenance and survival of neurons during development and adulthood. Both life phases make specific demands on the molecular and physiological properties of astrocytes, and most research projects traditionally focus on either developmental or adult astrocyte functions. In most brain regions, the generation of brain cells and the establishment of neural circuits ends with postnatal development. However, few neurogenic niches exist in the adult brain in which new neurons and glial cells are produced lifelong, and the integration of new cells into functional circuits represent a very special form of plasticity. Consequently, in the neurogenic niche, the astrocytes must be equipped to execute both mature and developmental tasks in order to integrate newborn neurons into the circuit and yet maintain overall homeostasis without affecting the preexisting neurons. In this review, we focus on astrocytes of the hippocampal dentate gyrus (DG), and discuss specific features of the astrocytic compartment that may allow the execution of both tasks. Firstly, astrocytes of the adult DG are molecularly, morphologically and functionally diverse, and the distinct astrocytes subtypes are characterized by their localization to DG layers. This spatial separation may lead to a functional specification of astrocytes subtypes according to the neuronal structures they are embedded in, hence a division of labor. Secondly, the astrocytic compartment is not static, but steadily increasing in numbers due to lifelong astrogenesis. Interestingly, astrogenesis can adapt to environmental and behavioral stimuli, revealing an unexpected astrocyte dynamic that allows the niche to adopt to changing demands. The diversity and dynamic of astrocytes in the adult DG implicate a vital contribution to hippocampal plasticity and represent an interesting model to uncover mechanisms how astrocytes simultaneously fulfill developmental and adult tasks.

## Introduction

The assembly of a highly complex organ such as the brain requires precise coordination between the formation of synaptic connection and integration of these synapses into functional circuits. Once developed, neural circuits need to be maintained and supported both structurally, and metabolically. Any alterations in assembly, but also in circuit homeostasis may have serious consequences and will most likely lead to neurodevelopmental, -psychiatric and -degenerative disorders [1–3]. Studies of neurons alone failed to understand how circuit plasticity is established and circuit balance is maintained. Astrocytes, a major glial cell type in the brain, are indispensable in the coordination of neural circuit assembly as well as their maintenance. These highly complex cells infiltrate the neuropil and instruct synapse formation, elimination and remodeling [4, 5]. As key components of neural circuits, astrocytes form the so-called “tripartite synapse” [6, 7] consisting of a neuronal pre- and a post-synapse, which are enwrapped by fine perisynaptic astrocytic processes (PAPs). PAPs are highly motile astrocytic structures that contact or abandon synapses in correlation with synaptic complexity and strength [8, 9]. Hence, astrocyte ensheathment appears to be a dynamic process, which is directly linked to neuronal activity [10], can be altered during development, and react to injury as well as to diverse physiological conditions [11–13]. The tight spatial relation enable neurons and astrocytes to bidirectionally communicate, including astrocytes responses to synaptic activity via neurotransmitter receptors [14, 15] as well as astrocytic release of so-called gliotransmitters to modulate neuronal activity [16–27]. Even though astrocytes are not electrically excitable themselves, they sense local synaptic activity through metabotropic and ionotropic receptors [28–30] and display Ca^2+^ transients that mirror neuronal activity [31–34]. Interestingly, both neuronal activity [35] as well as neuronal release of signaling component such as Sonic hedgehog can induce transcriptomic changes in astrocytes in vivo [36] suggesting that the interplay between neurons and astrocytes is dynamic on many levels.

In this review we will first summarize the latest findings on how astrocytes establish and maintain neural circuits. In the second part we will discuss how astrocytes in the adult dentate gyrus (DG), one of the few regions in which new neurons are generated lifelong [37], may contribute to DG function and plasticity.

## Astrocyte as key elements of circuit formation during development

The role of astrocytes in synaptogenesis was first demonstrated in vitro: While neurons were largely inactive in culture, the addition of astrocytes was sufficient to promote synapse formation and induced spontaneous neuronal activity across species [38–40]. Astrocytes release a variety of pro- and anti-synaptic molecular cues to orchestrate a dynamic process to fine-tune synapse numbers during circuit assembly (reviewed in [4, 41]). This has been best shown for glutamatergic excitatory synapses [42], but also recently for the formation of inhibitory GABAergic synapses [43].

During development, the expansion of astrocytic membrane domains parallels the generation and refinement of synapses within individual circuits [5, 42, 44–49]. In most brain areas, the first two weeks of postnatal development represent a period of massive synaptogenesis. Concomitantly, astrocytes express and secret a number of factors to initiate synapse formation. Cholesterol was the first identified molecule secreted by astrocytes (in complex with ApoE) for which a function in synaptogenesis could be revealed. By increasing the number of vesicles in the presynaptic terminals as well as their release probability, cholesterol boosts synaptic function [50, 51]. Also, astrocytic release of thrombospondins (TSP 1 and 2) promotes the formation of structurally normal but silent excitatory synapses [47]. Spinogenesis is then promoted by stimulating actin remodeling at the postsynaptic side by activating Rac1 [52]. The release of the innate immune molecule pentraxin3 (PTX3) promotes the functional maturation of excitatory synapses by inducing AMPA receptors’ clustering at the synapse [4, 44, 53, 54]. AMPA receptor clustering is further stimulated by neuronal pentraxin 1 receptor (NP1), which is induced by the release of Glypican 4 from astrocytes [53]. The secretion of Hevin by astrocytes bridges presynaptic neurexins and postsynaptic neuroligins, which induce the recruitment of PSD95 and NMDAR subunits at the synapse, an effect which is antagonized by SPARC through a yet unknown mechanisms [42, 48, 55]. Synapse maturation and stabilization are accompanied by replacing calcium-permeable AMPA glutamate receptors (AMPARs) by GluA2-containing calcium-impermeable AMPARs. This AMPAR-switch is mediated by Chordin-like 1 (Chrdl1) secreted by astrocytes in the cortex. Chrdl1 knockout mice as well as humans with mutations in Chrdl1 display enhanced plasticity and learning, suggesting that astrocytes promote GluA2-dependent synapse maturation, and limit synaptic plasticity at the end of development [56]. Many more molecules and mechanisms are included in the assembly of a functional synapse (reviewed in [57]), however, it is important to mention that not only secreted factors, but also contact-mediated signals between astrocytes and neurons are involved in synaptogenesis.

Once neural circuits have formed, they undergo crucial refinements to eliminate overproduction of synapses in an experience-dependent manner [58]. Here, the developing nervous system adapts to the onset of neuronal activity as a direct consequence of arriving environmental stimuli via sensory afferents [59–61]. These so-called “critical periods” are short windows during which neural circuit activity can modulate morphological properties of neurons leading to permanent changes to circuit structure and function [62]. Unnecessary and weak synapses are eliminated, while remaining inputs are strengthened, a process that continues but decelerates in adult stages [63]. This elimination is not only an intrinsic property of neurons, but heavily regulated by microglia and astrocytes, which remove excitatory and inhibitory synapses via two activity-dependent phagocytic receptors, MEGF10 and MERTK in the developing mouse visual system [64]. As demonstrated recently, astrocytes are indispensable for the closure of the critical periods in both sensory and motor system. In the visual cortex, astrocytic Connexin 30 (Cx30) promotes the maturation of inhibitory circuits through inhibition of extracellular matrix degradation via the RhoA-Rock pathway [65]. In Drosophila motor circuits, astrocytes close the critical period by cross talking to neurons via Neuroligin-Neurexin signaling. When astrocytes infiltrate the neuropile at late embryogenesis and enwrap motor neuron synapses, astrocyte-derived Neuroligin-2 interacts with neuronal Neurexin-1, which promotes dendritic stability by stabilizing microtubules [66]. Overall, decades of research on astrocytes have firmly established the undisputed role of astrocytes in the assembly, maturation and fine-tuning of synaptic connections to form functional neural circuits [67].

## Adult functions of astrocytes

During adult stages, astrocyte monitor, instruct and support neuronal activity in mature circuits [4, 68]. Astrocytes cover the brain in a tile-like non-overlapping organization, forming electrically coupled networks [69]. A single mouse astrocyte can contact over 100,000 synapses with their fine processes, and this number can further increase to up to 2,000,000 synapses in the adult human cortex [69, 70]. Perisynaptic astrocyte ensheathment serves to remove neurotransmitters released from the interstitial space to obviate extrasynaptic accumulation and neurotransmitter spill-over to neighboring synapses [71]. Excessive glutamate is rapidly transported into astrocytes via the glutamate transporters Glt1 and GLAST [72, 73], where it is converted into glutamine by the glutamine synthase and shuttled back to neurons. Through this mechanism, astrocytes limit glutamate induced excitotoxicity [74] and allocate new material for ongoing synaptic transmission [75]. Furthermore, astrocytes regulate the ionic balance at the synapse through numerous channels to maintain proper synaptic transmission [76, 77]. Through metabotropic and ionotropic receptors, astrocytes are able to sense local activity of synaptic circuits [78]. Receptor activation leads to the generation of dynamic Ca^2+^ transients in astrocytes [79], which in turn may induce secretion of gliotransmitters [40, 80, 81]. These neuroactive molecules signal directly back to the synapse to regulate proper basal synaptic transmission and modify neural plasticity [20].

Plasticity is a process in which neural circuits undergo structural and molecular changes in order to adapt their function in response to new environmental requirements. Historically, plasticity has been regarded predominantly from the neuronal perspective and only recently, the focus began to shift towards the non-neuronal part of the brain. A region, which has been fundamental in establishing mechanisms how astrocytes influence, regulate, and shape neuronal plasticity in the adult brain, is the hippocampus. The hippocampus is a fine-layered structure, involved in the formation of episodic memory [82]. Here, especially the Schaffer collaterals – Cornu Ammonis area 1 (CA1) synapses as well as mossy fiber - CA3 synapses have been extensively studied. Electrophysiology as well as stimulation paradigms have shown that bidirectional modifications can take place at the same synapse, leading to either long-term depression or long-term potentiation (LTD or LTP, respectively) as a direct consequence of neuronal activity. Many of the astrocyte-secreted factors described above (Hevin, SPARC, TNF-alpha) are involved in synapse-remodeling as well as in the regulation of LTP, LTD and a specialized form of plasticity called homeostatic scaling [83]. While LTP and LTD are important for rapid adjustment in the strength of individual synapses in response to correlated synaptic activity, homeostatic synaptic scaling entails uniform adjustments in the strength of all synapses of a neuron in response to changes in neuronal activity patterns [84].

Blocking of neuronal activity increases astrocytic expression and secretion of Interleukin-33 (IL-33), which lead to an increase in excitatory synapses and neurotransmission through synaptic recruitment of the scaffold protein PSD95. Blocking of IL-33 in turn impairs spatial memory formation in mice, indicating a negative-feedback signaling mechanisms in synaptic plasticity in CA1 pyramidal neurons [85]. Seminal work from the lab of Inbal Goshen revealed that activation of astrocytes, not neurons, induces de novo NMDA-dependent LTP in CA1, which enhances memory acquisition and promotes memory allocation [86]. During learning, the neuronal communication from CA1 to the anterior cingular cortex involves projection-specific functions of CA1 astrocytes, which affect remote, not recent, memory recall [87]. Recently, using real-time Ca^2+^ two-photon imaging in CA1 astrocytes of awake mice, Doron and colleagues showed that astrocytes can encode reward location in spatial contexts [88], thereby extending the astrocytes computational ability as well as their role in cognitive function. To establish stable long-term memory, the initial memory acquisition is followed by memory consolidation, a period in which synaptic plasticity occurs. The memory is then maintained through a process called memory retention, in which changes in neural circuits are preserved, and can be recalled by reactivation of the related circuits [89, 90]. Multiple receptors expressed in astrocytes transduce signals via the second messenger cAMP. Increasing cAMP levels in hippocampal astrocytes in vivo by photoactivation of the adenylyl cyclase facilitated memory formation, but intermitted memory retention. This modulation of memory was suggested to be mediated by the astrocyte-neuron lactate shuttle [91].

Likewise during development, synapses in the adult brain are constantly formed, but also eliminated. The elimination of synapses is a fundamental process in neuronal circuit refinement and homeostasis. Using fluorescent phagocytosis reporters, it has recently been shown that in the CA1 region of the hippocampus, especially excitatory synapses are eliminated by astrocytes expressing the phagocytic receptor MEFG10. Knockout of Megf10 in adult astrocytes leads to a reduction in synapse elimination, defective LTP and impaired formation of hippocampal memory [92]. Such a role has also been revealed in the cerebellum where engulfment of synapses by Bergmann glia frequently occurs upon motor learning. Pharmacological blockade of engulfment with Annexin V inhibits overnight improvement of motor adaption [93]. These findings highlight the key functions of astrocytes in synaptic plasticity, memory and learning in the adult brain.

### Astrocytes of the adult dentate gyrus need to fulfill both developmental and adult tasks

An area of the hippocampus, which is highly interesting in regard to plasticity, is the dentate gyrus (DG). The DG is the input area/relay station of the hippocampus-entorhinal cortex circuit and harbors a very specialized form of plasticity. Here, not only single synaptic connections are modified, but entirely new neurons are integrated into existing circuitries. The DG is one of the few so-called neurogenic niches in the adult brain, in which new neurons and glia are generated by radial glia-like neural stem cells (NSCs) lifelong. The adult NCS lineage consists of a stereotypic sequence of distinct developmental steps, in which a mostly quiescent radial glia-like NSCs divides in a self-renewing manner to generate actively proliferating intermediate progenitor cells (IPCs [94]); IPCs give rise to neuronally committed neuroblasts that exit the cell cycle to mature. The generation and integration of these new neurons into existing circuitries are central processes to hippocampal plasticity and involved in higher cognitive functions such as learning, memory processes, and mood regulation [95]. From the astrocyte’s perspective, this is highly interesting as DG astrocytes have to simultaneously fulfill both developmental and adult tasks to (i) establish new synaptic connections for newborn neuron integration (like during development), and (ii) to maintain synaptic connections established during development (adult brain function). Our group recently uncovered two interesting finding in regard to astrocyte in the adult DG: (i) DG astrocytes are heterogeneous based on molecular, morphological and functional properties [96]. Intriguingly, the distinct astrocyte subtypes are localized to specific compartments, thereby recapitulating the structural organization of the DG. (ii) Astrocytes in the adult DG locally proliferate to generate new astrocytes. This proliferative capacity is dynamic and can be adapted to environmental and behavioral stimuli [97]. In the second part of this review, we will discuss potential roles of astrocyte diversity and dynamics in adult DG’s plasticity.

## Astrocyte diversity

**Fig. 1:**
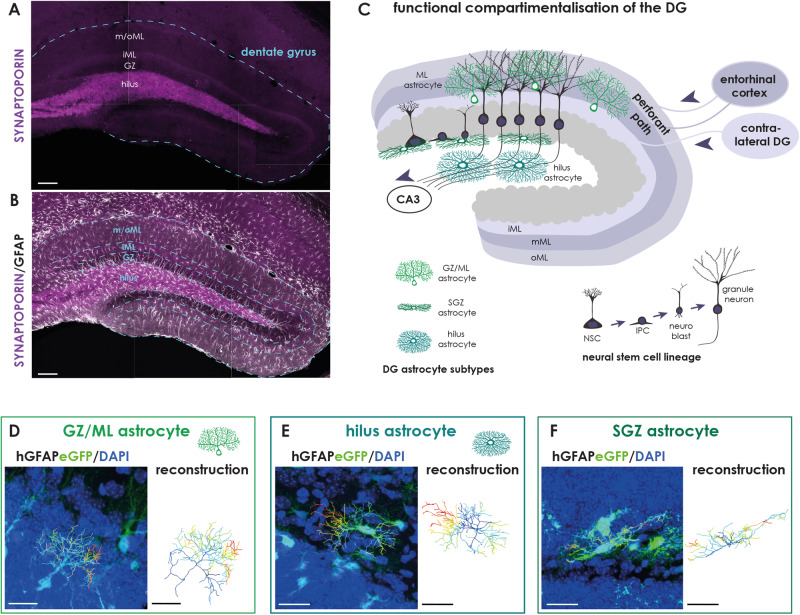
Astrocyte subtypes in the adult DG are associated to distinct functional compartments. **A** Confocal image of the adult DG (blue line) immunostained against SYNAPTOPORIN (magenta) illustrating the distinct layers (hilus, GZ granule zone, iML inner Molecular layer; m/oML medial and outer Molecular layer). **B** Astrocytes of the adult DG as revealed by GFAP immunostaining (white). **C** Schematic depicts the compartmentalization of the adult DG. Afferents from the entorhinal cortex form the medial and outer ML, while those from the contralateral DG form the inner ML. The neurogenic lineage (gray) consists of a neural stem cell (NSC) giving rise to actively proliferating intermediate precursor cells (IPCs), which generated neuroblasts (NBs). NBs subsequently mature to granule neurons. **D**–**F** Astrocytes reveal a subtype-specific morphology as determined by the expression of enhancedGFP (green) in brain slices of hGFAPeGFP transgenic animals (on the left). The eGFP signal was reconstructed in IMARIS (depicted on the right); different colors highlight branching order (dark blue=primary; light blue=secondary; turquoise=tertiary; green=quaternary; light green=quinary; yellow=septenary; orange=octonary; red = nonary). **D** Astrocytes of the upper DG compartments (ML and GZ) show a polarized morphology with one primary process emerging from the soma and fanning out into the ML with treetop-like branches. **E** Hilus astrocytes reveal a bushy morphology with several primary processes pointing in each direction. **F** SGZ astrocytes are smaller and stretch out one to two primary branches along the SGZ boarder. Scale bar = 100 μm (**A**, **B**) and 20 μm (**D**–**F**).

The adult DG is an integral portion of a larger functional brain system called the hippocampal formation. The source of most sensory information designated for the DG is the entorhinal cortex, which projects to the DG via fibers called the perforant path. Since this connection is unidirectional, the DG is considered to be the first instance in the processing of information that leads to the production of episodic memory. The information received from the entorhinal cortex ultimately conveys to the CA3 field of the hippocampus. The highly ordered laminar distribution of many DG inputs is coupled to a regular organization of the DG’s three principal cell layers (Fig. 1A–C). In its outer compartment, the molecular layer (ML), perforant path input impinges on glutamatergic granule cell dendrites. The cell bodies of granule neurons are densely packed in the second DG layer, the granule zone (GZ), which encloses the hilus, also called the polymorphic layer. The hilus is filled up by granule cells axons, called mossy fibers, projecting towards the CA3. The subgranular zone (SGZ) may represent a fourth layer of the DG as it constitutes the proliferative zone in which radial glia-like NSCs proliferate to constantly fill up the DG with new granule neurons. It is important to note that the trilaminar structure of the DG is common across all species studied, aside from size differences, with no substantial phylogenetic modifications [98, 99].

Our recent work uncovered an interesting link between astrocyte diversity and the laminar compartmentalization of the adult DG such that different DG layers are populated by distinct astrocyte subtypes. The association with a specific DG compartment is accompanied by a subtype-specific morphology and molecular profile, leading to subtype-specific physiological characteristics. Overall, our findings suggest that distinct DG astrocytes may exert compartment-specific functions, adding another layer of complexity to our understanding of the DG network composition. In the following we will zoom in onto distinct DG compartments to discuss the specific features and potential functions of the astrocytes located there in regard to the layer-specific cellular environment.

### Astrocytes located in the upper DG compartments (molecular zone and granule layer)

GZ and ML astrocytes reside in a predominantly neuronal environment, surrounded by cell bodies (GZ) and dendrites (ML) of granule neurons. We discuss our findings on both subtypes together since they have many common features, while being explicitly different from other DG astrocyte subtypes. The principle neuronal cell type of the DG is the glutamatergic granule neuron. Granule neuron cell bodies are densely packed within the GZ stabled on top of each other in 8–10 cell rows. A single proximal dendrite emerges from the soma, stretches through the GZ and extensively branches out in the ML to form a cone-shaped tree of spiny apical dendrites, ending distally at the fissure (suprapyramidal blade) or the ventricular surface (infrapyramidal blade). The innervation of granule neurons occurs specific to their cell parts: the soma and proximal shaft of the apical dendrite are innervated predominantly by pericellular plexus formed by inhibitory interneurons, mostly GABAergic basket cells. The excitatory input can be separated into connections with commissural-associational projections derived from mossy cells of the contralateral DG (inner third of the ML), and perforant path afferents from the medial and lateral entorhinal cortex terminating on the middle and outer third of the ML, respectively. Related to the morphology of granule neurons, GZ astrocytes acquire a polarized morphology characterized by a soma located in the GZ from which a primary process stretches into the ML where it fans out into numerous side branches to occupy a large territory (Fig. 1D). This stereotyped morphology ideally serves to cover many synapses between granule neurons and perforant path afferents. Astrocytes with their soma located in the inner ML look much alike GZ astrocytes, but have a shorter primary process. The further out astrocytes are located within the ML, the more their morphology shifts towards a more multipolar, “star-shaped” morphology with up to five primary processes.

Physiologically, a striking feature of upper DG layer astrocytes is their subtype-specific gap junction coupling. In general, astrocytes form electrically networks called syncytia, in which neighboring astrocytes are connected via gap junctions, which are formed by connexins. Electrical coupling mediated by gap junctions plays an important role in the generation of highly synchronous electrical activity and coordinates single astrocytes to function as a highly efficient system in brain homeostasis [100]. Injection of a membrane-impermeable dye that only spreads through gap junctions demonstrated that upper layer astrocytes form big syncytia, as revealed by the coupling length constant and cumulative somatic fluorescence intensity [96]. While this may not appear unexpected, the surprise was that ONLY upper DG layer astrocytes formed such syncytia, while astrocytes of the lower DG compartments (SGZ and hilus) showed a significantly weaker coupling strength, hence formed much smaller syncytia. Notably, the strength of gap junction coupling was reduced across the GZ, indicating that upper layer astrocytes do not couple to lower layer astrocytes. These almost exclusively intra-subtype syncytia formations support the idea that astrocytes, also on a network level, “respect” the laminar compartmentalization of the adult DG. This reminds of a finding in the somatosensory thalamus, where cellular domains called barreloids are the structural basis for somatotopic whisker representation. Here, barreloid borders are formed by uncoupled glial cells, which limit gap junction communication across adjacent barreloids [101]. The question is why GZ/ML astrocytes form large networks, while SGZ and hilus astrocytes are only weakly coupled to their neighbors. The integration into a syncytium allows neighboring astrocytes to tile with one another, yet maintain non-overlapping territories, and associates single astrocytes with millions of synapses with an expanded glial network. A recent in vivo study demonstrated that extensive astrocyte synchronization via gap junction coupling precedes neuronal synchronization in slow wave activity, a characteristic brain oscillation in sleep [102]. Considering that the perforant path is the relay station of the hippocampal circuitry, these data may indicate that synchronization of hippocampal neuroglial networks occurs mainly at the input area and is subsequently not required anymore for the connection of granule cells to CA3 pyramidal neurons. Which physiological consequences may arise from that for hippocampal network dynamics is an interesting topic for future investigations.

Another interesting aspect of astrocytes in the upper DG layers is their strategic position to regulate synapse formation and synaptic integration of newborn neurons into existing circuitries. Using a combination of serial-section immune-electron microscopy, confocal microscopy and electrophysiology, the lab of Nicolas Toni revealed that astrocytes remodel their processes to form functional perisynaptic processes on newly generated neurons in the DG [103]. The authors raised the hypothesis that astrocytes play a structural role in connecting the spines of newborn neurons by ensheathing preexisting axons. They could show that perisynaptic processes are already present when the first dendritic spines and axonal terminals appear in newly generated neurons. The astrocytic processes are very plastic and their motility seems to be coordinated with motility of the dendritic spines. In line with astrocyte structural plasticity upon neuronal activation described before [11], astrocytes can increase their coverage of excitatory synapses on dendritic spines during modified neuronal activity. The proportion of newborn and mature granule neurons covered by astrocytic perisynaptic processes is much higher in the DG compared to those found in Schaffer collateral [103]. The same group could also show that the formation of dendritic spines on newborn neurons as well as their functional synaptic integration is regulated by local DG astrocytes via vesicular release of the gliotransmitter D-serine [104]. These finding implicate an important role of astrocytes in the maturation and survival of newly generated neurons in the adult DG. Given that upper DG layer astrocytes are closely intertwined with the dendrites of newborn neurons, it is almost intuitive that they are the prime candidates to regulate dendritic spine integration, and it will be the content of future research to establish further mechanisms through which ML and GZ astrocytes mediate these processes.

### Astrocytes located in the lower DG compartments (SGZ and hilus)

The lower DG compartments comprise the SGZ and hilus, also known as the polymorphic layer in humans. Granule neurons give rise to distinctive unmyelinated axons, the mossy fibers. The name was determined by Ramón y Cajal because of the unusually large boutons that form synapses with CA3 pyramidal neurons. On their way to CA3, mossy fiber axons give rise to a distinct set of collaterals that form contacts with proximal dendrites of mossy cells [105] and basal dendrites on pyramidal basket cells. However, the vast majority of mossy fiber collaterals terminate on GABAergic interneurons. Astrocytes located in the hilus acquire a bushy morphology with four to five primary branches emerging in all directions from the soma and are in intimate contact to the mossy fibers. In contrast to the upper layer astrocytes, hilus astrocytes do not appear polarized and rather resemble fibrous astrocytes with whom they share the association to mainly axonal structures (Fig. 1E). As described already above, hilus astrocytes form small syncytia and are connected to only very few neighboring astrocytes. Interestingly, seemingly mature, morphologically elaborate astrocytes in the hilus frequently divide in the adult brain and keep their proliferation capacity up to old ages (21 months in mice; [97]). While we do observe local proliferation of astrocytes in all DG layers, the proliferation rate is much higher in lower layer astrocytes than in those residing to the upper layers. It is reasonable to speculate that there may be a link between proliferation capacity and syncytium integration of astrocytes subtypes, such that astrocytes which are less integrated into stable networks may have more freedom to divide. However, what is the cause and what are the consequences is difficult to assess. Another interesting feature of hilus astrocytes is their association with Alzheimer’s disease (AD). AD patients show a significant accumulation of tau protein in hilus astrocytes, and overexpression of 3R tau specifically in hilus astrocytes is sufficient to induce AD-like symptoms, such as neuronal dysfunction and memory deficits [106]. These observations highlight the importance of astrocyte subtype-specific features in neurodegenerative diseases.

The SGZ constitutes a single cell layer in which radial glia-like NSCs reside, which occasionally divide to give rise to newborn neurons via actively proliferating precursor cells. Hence, the SGZ is primarily characterized as the proliferative zone. Astrocytes located in the SGZ stretch out long horizontal processes (Fig. 1F), which are closely associated to radial-glial-like NSCs and cradled around clusters of proliferating IPCs and newborn neurons, implying a supportive, if not regulatory, function of SGZ astrocytes in the process of adult neurogenesis. In contrast to all other DG astrocyte subtypes, the morphology of SGZ astrocytes differ between mouse and humans with human SGZ astrocytes lacking those horizontal processes. This is interesting as adult neurogenesis appears frequently in the adult mouse brain, but is a rare event in the human DG [107–110]. These observations support the hypothesis that astrocyte morphologies (i) are directly influenced by their neighboring cellular and structural microenvironment and (ii) implicate potential functions of astrocyte subtypes. SGZ astrocytes revealed a much higher transcriptional rate than other astrocytes, with significantly upregulated genes associated to nervous system development, neurogenesis, regulation of neuronal differentiation and migration, signaling, cell-cell communication, and secretion [96]. Given their morphological and molecular fingerprints, we speculate that SGZ astrocytes maybe involved in the regulation of distinct steps of the neuronal lineage, such as NSC activation (or quiescence), proliferation of progenitors, fate decision of NSC progeny, and/or elimination of newly generated neurons.

Diversity raises the question whether or not functional subsets exists or whether all astrocytes fulfill all functions. Our findings on adult DG astrocytes indicate that astrocyte subtypes are equipped to fulfill specific functions as distinct local components in the hippocampal trisynaptic circuit. Despite a quite strong similarity on molecular levels, we speculate that astrocyte key functions such as synapse formation, elimination, maturation as well as synaptic homeostasis and modulation of synaptic transmission functions are not necessarily performed by all astrocytes within the DG. We rather think that functional set ups of astrocytes exist that locally act according to the needs of specific compartments. An important question is where this functional specialization arises from. Here, an educated guess would be that neurons (or neuronal structures) take over an instructive role in determining astrocyte identity as it has been already demonstrated on molecular and morphological levels [36, 111, 112]. This bona fide division of labor is already commonly accepted on interregional levels since many functions of astrocytes appear to be either region specific or enhanced in defined types of astrocytes. New straightforward criteria how to define astrocyte diversity would therefore be by defining the astrocytes interaction on circuit level, as suggested in a recent review by Benjamin Deenen [113].

## Astrogenesis and astrocyte dynamics

### Astrogenesis and astrocyte lineage progression

Despite the broad knowledge gained over the last decades on neurogenesis and oligodendrogenesis, our understanding of astrocytic origin and development is still limited. In this review we will not discuss in detail how astrogenesis is regulated on genetic and epigenetic levels (for review see [114]). Our aim is rather to sum up current knowledge on astrocyte lineage progression and dynamics. Nearly all astrocytes in the developing cortex derive from GFAP-expressing progenitors [115, 116]. Also in the developing DG, we could show that astrocytes derive from NESTIN-expressing NSCs, which generate the first astrocytes within the first postnatal week. Similar to the developing cortex [117], the enlargement of astrocytic compartment in the DG is not NSC-driven but mainly mediated through proliferation of local astrocytes [97]. Our results suggest a lineage model in which NSCs are at the apex, giving rise to (intermediate) astrocyte precursors, which locally divide to generate more astrocytes. This rather conserved lineage progression shared by all neural cell types evokes a number of important follow up questions: (i) how is expansion accomplished? (ii) how are appropriated astrocyte numbers accomplished? (iii) which mechanisms govern local astrocyte proliferation? (iv) How do newborn astrocyte mature? These questions have been difficult to address because of the lack of good markers to distinguish NSCs, astrocyte progenitors and mature astrocytes. Given the overall glial nature of NSCs, they express common astrocyte markers like GFAP, GLAST, GLT1. NSCs can be identified by the presence of a radial-glial process and the expression of NESTIN, which are both absent in proliferation astrocytes. However, the only criteria to distinguish proliferating astrocytes from seemingly mature ones is the expression of cell cycle markers, reflecting obviously a highly transient state. Temporal profiling of developing astrocytes in the spinal cord revealed stage-specific gene expression profiles [118]. This is in line with single cell RNA-sequencing data in the DG, where we identified a clear molecular separation of proliferating astrocytes from non-proliferating astrocytes [96]. Astrocytes contain a rather low number of transcripts compared to other neural cell types, such as neurons. In contrast, the most striking characteristics of proliferating astrocytes is their high transcriptional rate [96]. However, all our attempts to find antibodies to specifically label proliferating astrocytes failed so far. This can be due to various reasons, such as technical difficulties to get antibody stainings to work. Our data rather imply a well-known problem regarding most “-omics”: mRNA expression and protein levels can vary quite distinctly and many of the numerous transcripts in proliferating astrocytes are not translated to proteins [96]. In fact, cell type-specific “uncoupling” of transcription from translation has been reported already [119]. Neurons for example maintain a subset of mRNAs in a translational silent state and act “on demand” to intra- and extracellular signals, while NSCs translate abundant transcripts with little discrimination [119]. Astrocytes seem to apply yet another strategy: non-proliferating astrocytes contain a low number on transcripts with abundant protein expression, suggesting that proteins in mature astrocytes are rather stable and do not require a constant transcription-mediated turnover. On the other hand, proliferating astrocytes significantly upregulate transcription with no change in protein levels, which may represent a transient state in which dividing astrocytes are preparing themselves for the high demand of cellular building blocks required for cell division. Future research will be necessary to decipher the bigger biological picture from gene transcription to mRNA translation in astrocytes.

A highly interesting, at present state of knowledge, almost philosophical question is whether or not proliferating astrocytes are indeed progenitors in the classical sense or whether all astrocytes own the potential to divide in general. This question will not be easy to solve on a population level and by still-picture analysis. Instead, it requires long-term in vivo imaging of astrocytes in the DG to stochastically determine astrocyte proliferation at the single cell level. If astrocyte precursors exist, it will be important to identify molecular markers, which separates them from postmitotic cells, to functionally assess their properties and to gain access to potential mechanisms regulating lineage progression. If all astrocytes retain the potential to divide, it would be of the highest interest to identify both instructive and inhibitory signals that govern where, when and how often an astrocyte divides.

While we do understand more and more about how astrocytes are generated, a yet understudied process is how astrocytes mature. Astrocyte-astrocyte contact has been shown to inhibit proliferation and to induce the expression of genes associated with “mature” astrocytes [120]. Apart from direct contact, astrocyte maturation also requires the downregulation of signaling pathways such as EGFR and SHH signaling [121, 122]. For future studies, it is important to better define what astrocyte maturation means. Until now astrocyte maturation has been mostly assessed by morphological analysis and by the expression of “mature” astrocytic genes such as S100ß. While the development of fine astrocyte branches and perisynaptic processes as well as establishment of astrocyte territories represent important steps, we still lack substantial knowledge on how astrocytes mature at the physiological level. Is a mature astrocyte defined by its integration into astrocytic syncytia or by communication with other astrocytes by Ca^2+^ waves? Is it the expression and/or secretion of gliotransmitters? Or is maturation based on distinct metabolic features? To really address the maturation processes, we need to find answers to these questions.

### Astrocyte dynamics

**Fig. 2:**
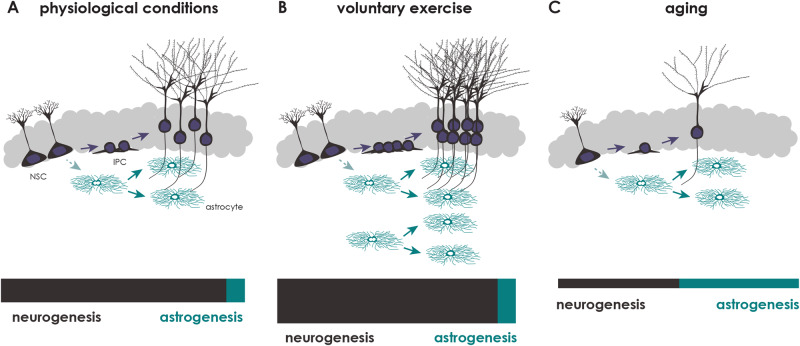
Ratios of neurogenesis and astrogenesis in the adult DG under different environmental condition and stimuli. **A**–**C** Schematics depict neurogenesis (dark gray) and astrogenesis (green). **A** Under physiological conditions, the vast majority of newly generated cells are neurons. In contrast to neurons, most adult-generated astrocytes derive from local astrocyte proliferation, and not from a direct NSC-division. **B** Voluntary physical exercise by running wheel significantly increases the number of newly generated neurons and astrocytes, but does not change the ratio between neuro- and astrogenesis in the adult DG. **C** From six months of age, the number of newborn neurons is strongly decrease, while new astrocytes are generated until much older ages, leading to a shift in the ratio towards astrogenesis.

In contrast to other non-neurogenic brain regions, astrogenesis in the DG does not stop after postnatal development but occurs until old ages [123–125]. Notably, the principle of astrogenesis is the same during development and in the adult DG. Throughout life, the generation of adult-born astrocytes is again mainly driven by local astrocyte proliferation (Fig. 2A), instead of astrogenic divisions of radial-glial-like NSCs [97]. Surprisingly, adult astrogenesis is a dynamic process, which can be stimulated by voluntary exercise [97]. Voluntary exercise represents the strongest known stimulus of adult neurogenesis and increases the generation of adult-born neurons by around 25-30% [126]. Astrogenesis increases at the same rate upon exercise leading to the maintenance of a fixed ratio between neurons and astrocytes in the adult DG [97] (Fig. 2B). These observations strongly indicate the existence of yet-to-be-identified signals/mechanisms to match the numbers of newborn astrocytes to those of newborn neurons. Upon aging, adult neurogenesis strongly decreases due to an exhaustion of the radial-glial-like NSC-pool together with detrimental effects on neuronal maturation [124, 127–130]. Interestingly, astrogenesis is still preserved and only significantly reduces at the much older age (21 months in mice) leading to a shift towards astrogenesis in aging animals (Fig. 2C). An obvious question is why the aging neurogenic niche still undertakes the effort to generate new astrocytes while almost no new neurons are generated. Based on our observations from single cell RNA-sequencing that astrocytes in the aging DG upregulate genes associated to synapse organization, axogenesis and axon guidance [97], it is tempting to speculate that astrocytes in the aging DG serve mainly to maintain synaptic connection and to stabilize existing neural circuitries to counteract aging-induced degenerative processes.

## Conclusion and future directions

Being the most numerous glial cell type in the brain it comes as no surprise that the functional roles of astrocytes far exceed the original notion that they were simply the ‘brain glue’. In fact, it is now appreciated how vital these cells are for the survival, integration, and maintenance of neurons, which in turn, leads to the overall proper functioning of the brain. From establishing and maintaining the blood brain barrier, to neurotransmitter uptake/recycling, to secretion of their own gliotransmitters, astrocytes are constantly at work to maintain proper homeostasis of the brain as a whole, as well as in region specific manners. Astrocytes perform their roles not only during the developmental time periods when neurogenesis is at its peak and the circuitry is being established, but also in post developmental stages, when the adult brain has distinctly separate demands for astrocytes. During development, astrocytic secreted signaling molecules promote the maturation of neurons and eventually lead to the establishment of both pre- and post- synaptic spines and synapses. These gliotransmitters are able to manipulate the synaptic machinery located within the spines, at the synapse level, continuing the neuronal maturation and initiate signal transduction and circuit formation. This process occurs in vast numbers during development, diminishes with age, and yet is still required in the adult brain to some degree. In the adult brain, astrocytes are vital for monitoring, instructing and supporting neuronal activity in mature circuits. Furthermore, astrocytes are crucially involved in synaptic plasticity, the process in which the synaptic strength can be modified leading to either LTD or LTP. An interesting exception to the functional separation of developmental and adult astrocytic task are the neurogenic niches of the adult brain. Here, astrocytes must be able to foster both mature and developmental environments in which established and newborn neurons can function and survive [131]. In this review we discuss recent findings on astrocyte dynamics and diversity, which may help to understand how astrocytes are able to accomplish these tasks.

Firstly, the astrocytic DG compartment is specifically dynamic, as astrogenesis occurs lifelong and the number of newborn astrocytes can be adapted to certain environmental stimuli. The integration of newborn neurons in the adult DG is a key component of hippocampal plasticity and it will be interesting to elucidate the functional role of astrocyte dynamics on this process. Secondly, it is now known that the astrocytes residing in distinct compartments of the DG demonstrate distinct features morphologically, molecularly and physiologically. Both the functional association to distinct neuronal structures as well as the spatial segregation may enable astrocyte subtypes to stably integrate new adult-born neurons, and yet to maintain overall homeostasis without affecting preexisting neurons. While the data on DG astrocyte diversity and dynamics represent the groundwork, much work has to be done in the future to understand the mechanisms behind the dual role of astrocytes in the adult DG. For that, it will be important to further analyze the physiological roles of astrocytes in other brain regions to fully understand where and how astrocytes maintain brain function and homeostasis. Elucidating the exact roles of neurogenic niche astrocytes, and astrocytes from other brain regions/structures, will be crucial to uncovering specific mechanisms associated with several neuropsychiatric and degenerative disorders including Autism Spectrum Disorder, Tourette’s syndrome, Schizophrenia, and Huntington’s disease, just to name a few. Understanding what specific roles astrocytes play in these contexts may allow for alternative and/or improved therapeutic approaches to tackle these disorders. While our understanding and knowledge of astrocytes has vastly improved over the last few decades there are still many questions and unknowns that need to be addressed. The more we discover about these diverse and dynamic glial cells, the more questions arise.
